# Respiratory-Gated Proton Beam Therapy for Hepatocellular Carcinoma Adjacent to the Gastrointestinal Tract without Fiducial Markers

**DOI:** 10.3390/cancers10020058

**Published:** 2018-02-21

**Authors:** Miu Mizuhata, Shigeyuki Takamatsu, Satoshi Shibata, Sayuri Bou, Yoshitaka Sato, Mariko Kawamura, Satoko Asahi, Yuji Tameshige, Yoshikazu Maeda, Makoto Sasaki, Tomoyasu Kumano, Satoshi Kobayashi, Kazutaka Yamamoto, Hiroyasu Tamamura, Toshifumi Gabata

**Affiliations:** 1 Proton Therapy Center, Fukui Prefectural Hospital, Fukui city, Fukui 910-8526, Japan; shigerad@staff.kanazawa-u.ac.jp (S.T.); khf05712@nifty.com (S.S.); sayu.b4242@gmail.com (S.B.); y-satou-xn@pref.fukui.lg.jp (Y.S.); y-tameshige-af@pref.fukui.lg.jp (Y.T.); y-maeda-ce@pref.fukui.lg.jp (Y.M.); m-sasaki-hl@pref.fukui.lg.jp (M.S.); k-yamamoto-7m@Pref.fukui.lg.jp (K.Y.); h-tamamura-8e@pref.fukui.lg.jp (H.T.); 2 Department of Radiotherapy, Kanazawa University Hospital, Kanazawa city, Ishikawa 920-8641, Japan; t.kumano@staff.kanazawa-u.ac.jp; 3 Department of Radiology, Nagoya University Graduate School of Medicine, Nagoya city, Aichi 466-8560, Japan; mkawamura@med.nagoya-u.ac.jp; 4 Department of Radiology, University of Fukui, Fukui City 910-1193, Fukui, Japan; asahis@u-fukui.ac.jp; 5 Department of Radiology, Kanazawa University, Kanazawa City, Ishikawa 920-8641, Japan; satoshik@staff.kanazawa-u.ac.jp (S.K.); tgabata@icloud.com (T.G.)

**Keywords:** proton beam therapy, hepatocellular carcinoma, 4D-CT, gastrointestinal tract, respiratory-gated irradiation

## Abstract

The efficacy of proton beam therapy (PBT) for hepatocellular carcinoma (HCC) has been reported, but insertion of fiducial markers in the liver is usually required. We evaluated the efficacy and toxicity of respiratory-gated PBT without fiducial markers for HCC located within 2 cm of the gastrointestinal tract. From March 2011 to December 2015 at our institution, 40 patients were evaluated (median age, 72 years; range, 38–87 years). All patients underwent PBT at a dose of 60 to 80 cobalt gray equivalents (CGE) in 20 to 38 fractions. The median follow-up period was 19.9 months (range, 1.2–72.3 months). The median tumor size was 36.5 mm (range, 11–124 mm). Kaplan–Meier estimates of the 2-year overall survival, progression-free survival, and local tumor control rates were 76%, 60%, and 94%, respectively. One patient (2.5%) developed a grade 3 gastric ulcer and one (2.5%) developed grade 3 ascites retention; none of the remaining patients developed grade >3 toxicities (National Cancer Institute Common Terminology Criteria for Adverse Events ver. 4.0.). This study indicates that PBT without fiducial markers achieves good local control without severe treatment-related toxicity of the gastrointestinal tract for HCC located within 2 cm of the gastrointestinal tract.

## 1. Introduction

Hepatocellular carcinoma (HCC) is one of the most important cancers worldwide because of its generally poor prognosis [1,2]. In addition to radiotherapy, the treatment strategies for HCC include surgery, transcatheter artery chemoembolization, radiofrequency ablation (RFA), and liver transplantation [3,4]. Radiation therapy has historically been considered ineffective for treatment of HCC. Because the liver is a radiosensitive organ, it is difficult to achieve a sufficient irradiation dose to treat HCC [5,6]. Proton beam therapy (PBT) for HCC has recently been reported to achieve good local control and less toxicity [7,8,9].

In contrast, the gastrointestinal (GI) tract is a serial organ that is sensitive to radiation [6]. A high dose of radiation therapy may cause severe damage [10]. Therefore, HCC adjacent to the GI tract should be carefully treated using the high-precision irradiation technique. For high-accuracy irradiation, Tsukuba University developed a respiratory gating system with fiducial markers and reported good treatment outcomes [8,11]. Furthermore, a four-dimensional computed tomography (4D-CT) technique was recently used to analyze the respiratory motion of the target and was shown to be useful for high-accuracy radiotherapy [12,13].

In this study, we used 4D-CT planning to measure tumor motion and retrospectively assessed the outcome of treatment for HCC adjacent to the GI tract without fiducial markers using breathing-synchronized irradiation.

## 2. Results

Of the 40 patients included in this study, 38 completed PBT according to the treatment protocol (Table 1). One of the two who did not complete the protocol had gastric hemorrhage with an ulcer treated by transfusion (grade 3), and PBT was finished earlier than the planned protocol (70 cobalt gray equivalents (CGE) in 35 Fr). The other patient developed uncontrollable ascites (grade 3) and also finished PBT earlier than the planned protocol (52.8 CGE in 24 Fr). Of the 40 cases, 22 were replanned and three were replanned twice. In total, we performed 25 replannings. Furthermore, three patients underwent unexpected replanning because of an unpredictable change in the GI tract position or shape at the time of 5-, 6-, or 10-fraction treatment (Table 2).

All patients were followed up until death or December 2017. The median follow-up time was 19.9 months (range, 1.2–72.3 months). The median survival time was 20.8 months. The overall survival (OS) rates after 1 and 2 years were 86% (95% CI, 75%–98%) and 76% (62%–91%), respectively. Of all potential prognostic factors in the univariate analysis, age, sex (female), number of tumors, and PS were associated with OS. In the multivariate analyses using Cox regression analysis, sex (female) and PS were significantly associated with good OS (Table 3).

**Figure 1:**
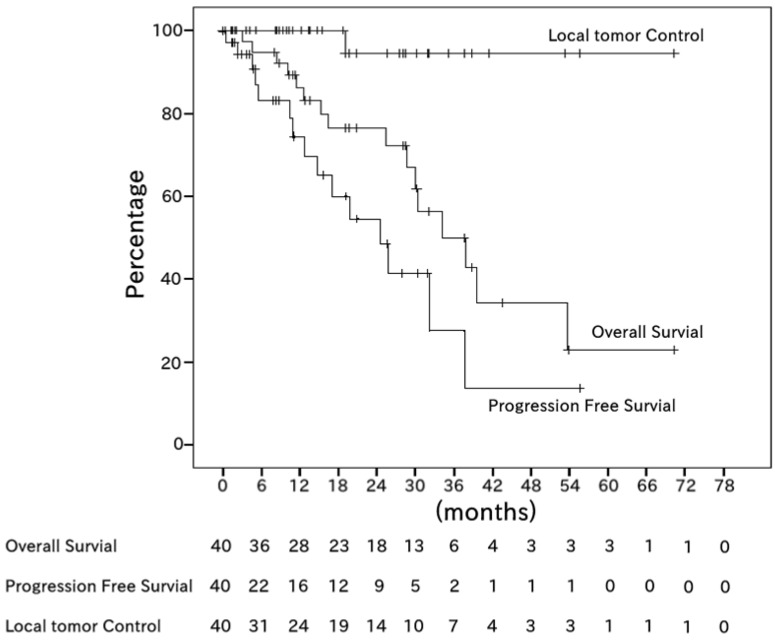
Kaplan–Meier estimates of overall survival (OS), progression-free survival (PFS), and local tumor control (LTC) among all 40 patients. The median OS period was 20.8 months (range, 1.3–72.3 months). The median PFS period was 9.8 months (range, 0.0–57.2 months). The 2-year OS, PFS, and LTC rates were 76%, 60%, and 94%, respectively.

The progression-free survival (PFS) rates after 1 and 2 years were 70% (95% confidence interval (CI), 55–86%) and 60% (42–79%), respectively. The local tumor control (LTC) rate after 2 years was 94% (95% CI, 83–100%) (Figure 1).

With respect to acute treatment-related toxicity, one patient with a bleeding gastric ulcer (grade 3) and another patient with uncontrollable ascites (grade 3) were unable to complete treatment. No patients had any other grade >3 severe acute toxicities; only skin reactions were confirmed (grade 1 or 2). With respect to late toxicity, one patient had GI bleeding (grade 2), and one had a rib fracture with pain (grade 2). No patients had grade >3 severe late treatment-related toxicity, including GI bleeding or perforation.

**Figure 2:**
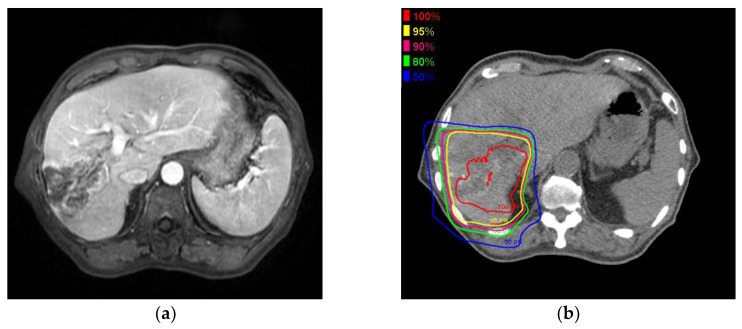

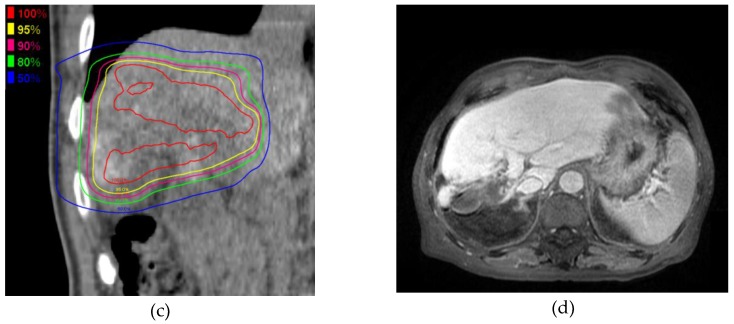
A 6.3 cm hepatocellular carcinoma (HCC) was treated by proton beam therapy (PBT) in a patient with Child–Pugh class A liver disease and right portal vein tumor thrombosis. (**a**) Early enhancement of the HCC was shown in the arterial phase of gadolinium ethoxybenzyl diethylenetriamine pentaacetic acid-enhanced dynamic magnetic resonance imaging (Gd-EOB-MRI); (**b**) The isodose lines were shown on planning CT (axial view); (**c**) This tumor was close to the colon (coronal view); (**d**) HCC was shown in the arterial phase of EOB-MRI 2 years after PBT.

Figure 2 shows the MRI and PBT planning results of a successfully treated 64-year-old man with a large HCC in the right hepatic lobe (Figure 2a). He was treated with PBT at 80 GCE in 25 fractions (Figure 2b,c). The tumor shrank and was controlled 2 years after PBT (Figure 2d). He showed no severe complications during a 2-year follow-up.

## 3. Discussion

### 3.1. Treatment Effect

A particle beam has different physical characteristics from a photon beam and can be useful for high-dose irradiation to HCC with a decreased background liver dose compared with photon therapy [14,15,16]. Many cases of good local control by particle beam therapy for HCC have recently been reported [17,18,19]. In PBT for HCC, Fukumitsu et al. [18] reported a 2-year local control rate of 87.8%. In addition, Kawashima et al. [19] reported a 2-year local control rate of 96%. Considering that the GI tract is susceptible to irradiation damage, irradiation doses to the GI tract should be reduced. In particle beam therapy, Abe et al. [20] reported that a carbon ion beam can reduce the GI tract dose more than can stereotactic body radiotherapy. Additionally, Nakayama et al. [21] performed PBT for HCC adjacent to the GI tract and reported good control and less toxicity with fiducial markers. In the present study, the 2-year local control rate was 94%, which is almost the same as in the above study.

Age, sex (female), number of tumors, and PS were associated with OS in the univariate analysis, while sex (female) and PS were associated with OS in the multivariate analysis (Table 3). Komatsu et al. [22] analyzed the factors associated with OS in 150 patients with HCC and reported that age, PS, and Child–Pugh classification significantly influenced OS in the univariate analysis. Tangkijvanich et al. [23] reported that among patients with HCC, female patients tended to have higher survival rates than male patients, but sex was not a predictor of patient survival. The number of tumors (solitary or not) is clinically important and an effective prognostic factor for OS. These clinical prognostic factors for OS in the present study are comparable with those in previous reports [9,24,25,26].

### 3.2. Adverse Effects

**Figure 3:**
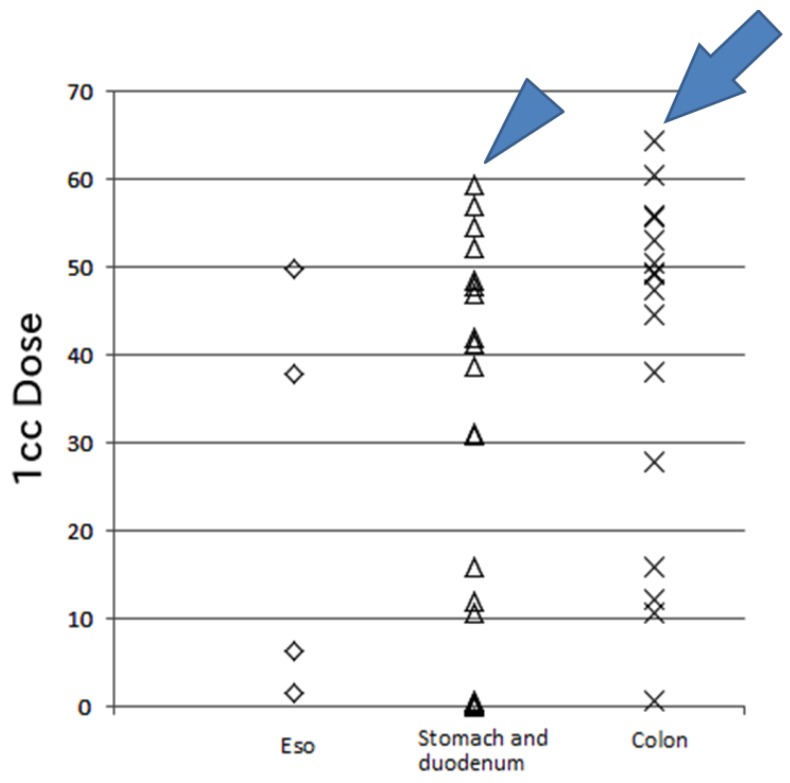
The 1-cc dose of the gastrointestinal tract. One case (arrowhead) involved a high dose (59.3 CGE) per 35 fractions to the stomach and duodenum, resulting in gastric hemorrhage with an ulcer treated by transfusion (grade 3). Another case (arrow) involved a high dose (64.4 CGE) per 20 fractions to the transverse colon without acute or late toxicity during the 3-year follow-up. Eso: esophagus, CGE: cobalt gray equivalent.

If the organs at risk are close to the irradiation field, more precise treatment is necessary, especially for organs affected by respiratory motion. In previously reported studies of PBT for HCC, fiducial markers were used for treatment [7,8,9,21,22,24]. Nakayama et al. [21] reported that 47 patients with HCC close to the GI tract were treated with PBT using metallic fiducial markers and 4 patients had grade 2 or 3 GI bleeding. In two of our patients, treatment was discontinued due to acute toxicity. The first patient had grade 3 gastric hemorrhage at the end of treatment (70 CGE in 35 Fr); this patient was irradiated at 59.3 CGE in 35 Fr (the 1-cc dose), which is the stomach tolerance dose (Figure 3) [6]. However, patients with liver cirrhosis are at risk of gastric bleeding, and this patient had a history of a gastric ulcer. In such cases, the irradiation dose should be as low as possible. After our experience with this patient, we attempted to reduce the 1-cc dose of the stomach and duodenum to <50 CGE, equivalent to 2 gray. Late gastric toxicity did not appear in this patient’s clinical course, and the HCC was controlled for 3 years.

The second patient who discontinued treatment was a 73-year-old woman with hepatitis C-related liver cirrhosis with bile duct invasion (Child–Pugh B; gross tumor volume, 252 mL; liver volume, 1320 mL). She stopped treatment due to ascites at the time of 52.8 CGE/24 Fr irradiation. Patients approaching a state of decompensated liver cirrhosis with a large target volume should be carefully treated and are at risk of developing ascites due to acute liver damage.

One patient with a high dose at the colon was a 38-year-old woman with hepatitis C-related liver cirrhosis (Child–Pugh class A) who developed HCC after transcatheter artery chemoembolization and RFA, and she was treated by PBT (76 CGE in 20 Fr) (Figure 3). Fortunately, she did not develop severe complications throughout a 3-year follow-up. However, the smallest dose possible should be administered to the colon considering the tolerance dose of this organ [6].

In this study, we attempted to reduce the GI tract dose by frequent performance of diagnostic imaging (CT or MRI) and application of adaptive PBT. No patients had severe late treatment-related toxicity, including GI bleeding and perforation.

### 3.3. Fiducial Markers

In this study, we did not use fiducial markers to achieve less invasive PBT. Fiducial marker insertion to the liver is safe, but in rare cases there is a risk of complications [27,28]. In the present study, most patients had inoperable disease, requiring us to avoid or reduce the risk as much as possible. Without insertion of fiducial markers into the liver, the diaphragm was used as the marker in this technique, and the daily inter-fractional error of the diaphragm in the craniocaudal direction was adjusted by the onboard imaging system. Balter et al. [29] and Yang et al. [30] reported that the diaphragm is an acceptable anatomic landmark for radiographic estimation of liver movement. Furthermore, in our respiratory gating method, the gate width with a duty cycle of about 20% (17–25%) is narrower than that obtained with the general method with a duty cycle of 20% to 40% [31]. The merit of a narrow gate is minimization of respiratory motion; however, the treatment time increases. For some patients, the treatment time was about 30 to 40 minutes because of the irregular respiratory rhythm.

We performed 4D-CT analysis the motion of the target, respiratory gating irradiation with a narrow gate width, frequent checks of changes in the GI tract, and adaptive PBT. Our results are consistent with those in other reports of PBT for HCC with fiducial markers in terms of LTC and adverse effects. Our treatment method is considered useful and safe.

The limitations of the present study include its retrospective design, small number of patients, and short follow-up period. Further prospective studies are necessary to assess a greater number of patients for a longer follow-up period.

## 4. Patients and Methods

### 4.1. Patients and Clinical Examination

This retrospective study was approved by the research ethics committee of our institution, and written informed consent for this study was waived because of its retrospective nature. From March 2011 to December 2015, 144 consecutive patients were treated at our institution. The inclusion criteria were (1) a medically inoperable condition considered difficult to control with RFA or patient refusal to undergo surgery or RFA, (2) an Eastern Cooperative Oncology Group performance status (PS) of 0 to 2, (3) hepatic function characterized by a Child–Pugh score of ≤10, (4) no uncontrolled ascites, (5) no extrahepatic metastasis, and (6) HCC adjacent to the digestive tract (≤2 cm distant of the digestive tract). Forty patients were considered eligible for analysis (Table 1). HCC was pathologically or clinically diagnosed by means of early nodular staining regarding the arterial enhancement and venous washout on dynamic CT and/or magnetic resonance imaging (MRI) and using serum levels of tumor markers (α-fetoprotein and des-γ-carboxyprothrombin) [4].

### 4.2. PBT Planning 

Patient setup, imaging planning, and use of the respiratory gating system were previously described [8,32,33]. A respiratory-synchronized 4D-CT system (Aquilion LB TSX-201A; Toshiba Medical Systems Co., Tochigi, Japan) with a respiratory gating system (Anzai Medical Co., Tokyo, Japan) was used under the following conditions: tube voltage, 120 kV; tube current, 200 to 300 mA; rotation time, 500 ms; and breathing synchronization using the 4D helical scan method. The patient was asked to perform stable breathing at a rate of 10 to 15 breaths/min using a metronome to maintain the appropriate rhythm. Patients with HCC close to the upper GI tract were examined in a fasting state. The reconstruction conditions of the CT images were as follows: slice thickness, 2 mm; slice interval (gap), 0.4 mm; and field of view set to match the physique of the patient.

The target delineation method was previously described [8]. Targets were contoured on the end of the exhalation phase of the 4D-CT image. The internal target volume was determined as the clinical target volume plus a 5- or 10-mm margin from the 4D-CT analyses. The internal margin was customized based on the amount of respiratory-induced motion visualized in the gate width for 1 s; a duty cycle of about 20% was centered at end-expiration [31]. The planning target volume was determined as the clinical target volume plus a 5-mm margin in all directions using a radiation treatment planning system with a proton pencil beam algorithm (XiO-N; Elekta, Mitsubishi Electric Corporation, Kobe, Japan). The beam angles were selected according to the following protocol: The treatment plan mainly involved more than two ports containing the perpendicular directions, and the beam directions were mainly selected to avoid the GI tract while cutting the radiation field using patient collimators [21]. Furthermore, patients with HCC in contact with the GI tract underwent replanning after 30 to 40 CGE irradiation with a cone-down boost (Table 2).

Additionally, the treatment plan was determined according to the following protocol: as large as the percentage volume of the non-irradiated normal liver [34], as low as the maximum exposure dose of the adjacent GI tract, and as low as the maximum 1-cc exposure dose in the adjacent GI tract [35] (Table 4). The total dose at the isocenter was prescribed to cover 95% of the planning target volume. The total dose for the target and organs were evaluated by rigid fusion techniques using commercially available software (MIM Maestro; MIM Vista Corp., Cleveland, OH, USA).

### 4.3. PBT

The PBT system used proton beams ranging from 150 to 230 mega-electron volts (MeV) generated through a linear accelerator and synchrotron. The 3D conformal proton dose distributions were formed based on a broad-beam method involving a couple of wobbling electromagnets, a scatter plate, a range modulator, and a multi-leaf collimator and in which the patient’s collimator and bolus were practically used. The patient’s collimators and boluses were custom-made to conform the beams to the treatment planning data (Mitsubishi Electric Corporation).

Nine protocols for respiratory-gated proton therapy (52.8–80.0 CGE in 20–38 fractions using 150-, 190-, or 230-MeV proton beams) were implemented during the study period using an irradiation schedule of 5 fractions per week (Table 2). The radiation dose was prescribed in CGE using a relative biological effectiveness value of 1.1 based on our preclinical experiments.

These protocols were based on previously reported studies [7,8,21,24]. To reduce the GI tract dose, patients with HCC close to the upper GI tract were treated in a fasting state. Daily irradiation was performed using more than two ports (with the exception of the plan using a one-port beam). The respiratory gating was configured for a beam-on cycle centered at end-exhalation for 1 s (duty cycle of about 20%). In this system, we can adjust the respiratory window; the beam irradiation is automated. If the patient’s respiratory rhythm becomes irregular or the patient begins coughing, we can push the button and easily avoid irradiation during the respiratory window.

Our image-guided radiation therapy system contains a biplane X-ray system with a full six-axis adjustability couch for the patient. We can correct setup errors matching to the vertebral body using the X-ray system in the anterior–posterior and right–left directions. The kV X-ray fluoroscopy can directly show real-time movement of the diaphragm and gas in the GI tract. Differences can be compared and the distance measured in the craniocaudal direction between the diaphragm contour at the time of planning and the diaphragm position in the deep aspiration phase onboard. We adjust the daily error of the diaphragm position at end-exhalation to plan the position of the diaphragm onboard by moving the treatment couch in the craniocaudal direction.

In the present study, we evaluated the target and GI tract by autoactivation imaging using positron emission tomography–CT. The data acquisition started 5 to 10 minutes after proton beam irradiation to assess the irradiated area on the first day of PBT [36]. Differences in the position or shape and total dose of the target or GI tract were evaluated by rigid fusion techniques using MIM Maestro. If the GI tract received high-dose proton beam irradiation by a change in the position or shape, we re-established the treatment plan. Furthermore, we evaluated the target and GI tract by CT and MRI at 15-fraction irradiation (3 weeks after beginning treatment) for adaptive treatment. If the high-dose irradiation was on the GI tract, the treatment plan was adaptively changed.

### 4.4. Follow-Up and Toxicity Evaluation

Abdominal CT and MRI examinations were performed at each follow-up and analyzed to determine the treatment effect (every 3 months after completion of PBT for the first 3 years, once every 6 months for the following 3–5 years, and annually thereafter). Tumor local control was assessed by simply determining if tumor regrowth was present using CT and MRI. Toxicity was graded according to the Common Terminology Criteria for Adverse Events ver. 4.

### 4.5. Statistical Methods 

The OS, PFS, LTC rates were analyzed using the Kaplan–Meier method, and a log-rank test was performed to evaluate statistical differences between two categories. OS, PFS, and LTC periods were calculated from the date of PBT start to the date of death or latest follow-up. Multivariate analyses were performed using a Cox regression analysis to identify the most significant independent prognostic factors. The following clinical variables were assessed using multivariate analysis: sex (female), age, chronic liver disease with viral infection, alcoholic liver, Child–Pugh class, operability, PS (0, 1/2), T classification, prior treatment, number of tumors (solitary or not), whole liver volume, tumor size, and gross tumor volume. The hazard ratio and 95% CI were calculated for each independent factor. All statistical analyses were performed using IBM SPSS version 20.0 software (IBM Corp., Armonk, NY, USA). A *p*-value of <0.05 was considered statistically significant.

## 5. Conclusions 

PBT for HCC located within 2 cm of the GI tract without fiducial markers at our institution is a safe and effective treatment without severe complications. Further investigation and long-term follow-up is necessary.

## Figures and Tables

**Table 1:** Patient and tumor characteristics.

Characteristics	*n*
Patients	40
Gender, male/female	28/12
Median age (range), years	72 (38–87)
PS 0,1/2	38/2
Median tumor size (range), mm	37 (11–124)
<50 mm/50–100 mm/>100 mm	27/8/5
Chronic hepatitis HCV/HBV/alcoholic/NASH/none	15/5/10/1/9
Child Pugh A/B	28/12
Tumor thrombus PV/HV/bile duct	12/2/10
Prior treatment TACE/RFA/PEIT/surgery	16/11/1/8
Operable / inoperable	4/36
Comorbidities anticoagulation/esophageal varices/history of GI bleeding or ulcers/none	2/9/3/26
T1/2/3/4	10/12/18/0
Solitary/multiple	10/30
Median GTV volume (range), cm^3^	21.4 (1.5–882.9)
Median liver volume (range), cm^3^	1259.2 (554.9–2198.6)
The GI-tract close to the tumorEsophagus/stomach and duodenum/colon	4/20/16

PS: performance status, HCV: hepatitis C virus, HBV: hepatitis B virus, PV: portal vein, HV: hepatic vein, TACE: transcatheter arterial chemoembolization, RFA: radiofrequency ablation, PEIT: percutaneous ethanol injection therapy, GTV: gross tumor volume, GI: gastrointestinal.

**Table 2:** Treatment protocols.

Total Dose(CGE)	Number of Fractions	Dose per Fractions	Equivalent Total Doses(2Gy/fraction)		Cases	The Number of Times of the Replanned Cases (number of cases replanned twice)	Fractions at Replanned (cases)
			α/β = 10	α/β = 3			
80.0	25	3.2	88.0	99.2	1	-	-
76.0	38	2	76	76	5	6(1)	5(1),20(3),30(2)
76.0	20	3.8	87.4	103.4	17	3	6(1),10(1),12(1)
74.8	34	2.2	76	77.8	3	3(1)	10(1),18(1), 20(1)
70.4	32	2.2	71.6	73.2	8	8	14(1),20(1),22(6)
70.0	35	2	70	70	3	4(1)	15(1),20(1),25(1), 30(1)
67.5	25	2.5	65.1	68.8	1	-	-
66.0	30	2.2	67.1	68.6	1	1	23(1)
52.8	24	2.2	53.7	54.9	1	-	-

Underlined text indicates unexpected replanning; CGE: cobalt gray equivalent.

**Table 3:** Variables affecting overall survival.

Variables	*p*-Value	HR	HR 95% CI	
			Lower	Upper
Age	0.232	1.034	0.979	1.093
Tumor number	0.912	1.096	0.216	5.561
sex (female)	0.003	11.903	2.319	61.088
PS	0.006	38.858	2.797	539.897

HR: hazard ratio, CI: confidence interval, PS: performance status.

**Table 4:** Gastrointestinal tract dose.

Organ	Distance (mm)	Max Dose (CGE)	1-cc Dose (CGE)	*n*
Esophagus	7(0–18)	43.4(15.3–54.4)	22.1(1.5–49.7)	4
Stomach and duodenum	11(4–19)	48.8(0.6–70.6)	34.9(0–59.3)	20
Colon	9(0–17)	53.4(12.9–71.6)	48.4(0.5–64.4)	16

Data are presented as median (range). CGE: cobalt gray equivalent.
